# SYMBIOmatics: Synergies in Medical Informatics and Bioinformatics – exploring current scientific literature for emerging topics

**DOI:** 10.1186/1471-2105-8-S1-S18

**Published:** 2007-03-08

**Authors:** Dietrich Rebholz-Schuhman, Graham Cameron, Dominic Clark, Erik van Mulligen, Jean-Louis Coatrieux, Eva Del Hoyo Barbolla, Fernando Martin-Sanchez, Luciano Milanesi, Ivan Porro, Francesco Beltrame, Ioannis Tollis, Johan Van der Lei

**Affiliations:** 1 EMBL-European Bioinformatics Institute, Wellcome Trust Genome Campus, Hinxton, Cambridge, CB10 1SD, UK; 2 Erasmus University Medical Center, Postbus 1738, 3000 DR, Netherlands; 3 Institut National de la Santé et de la Recherche Médicale (INSERM), Rennes, France; 4 Ministry of Education and Science, Paseo de la Castellana 160, Madrid, Spain; 5 Institute of Health "Carlos III", Carretera Majadahonda a Pozuelo Km. 2, 28220 Majadahonda (Madrid), Spain; 6 Institute for Biomedical Technologies (CNR-ITB), Via Fratelli Cervi 93, 20090 Segrate (MI), Italy; 7 National Research Council (CNR), Piazzale Aldo Moro, 5-00185 Roma, Italy; 8 Foundation for Research and Technology – Hellas (FORTH), Institute of Computer Science, P.O. Box 1385, GR-711 10 Heraklion, Crete, Greece

## Abstract

**Background:**

The SYMBIOmatics Specific Support Action (SSA) is "an information gathering and dissemination activity" that seeks "to identify synergies between the bioinformatics and the medical informatics" domain to improve collaborative progress between both domains (ref. to ). As part of the project experts in both research fields will be identified and approached through a survey. To provide input to the survey, the scientific literature was analysed to extract topics relevant to both medical informatics and bioinformatics.

**Results:**

This paper presents results of a systematic analysis of the scientific literature from medical informatics research and bioinformatics research. In the analysis pairs of words (bigrams) from the leading bioinformatics and medical informatics journals have been used as indication of existing and emerging technologies and topics over the period 2000–2005 ("recent") and 1990–1990 ("past"). We identified emerging topics that were equally important to bioinformatics and medical informatics in recent years such as microarray experiments, ontologies, open source, text mining and support vector machines. Emerging topics that evolved only in bioinformatics were system biology, protein interaction networks and statistical methods for microarray analyses, whereas emerging topics in medical informatics were grid technology and tissue microarrays.

**Conclusion:**

We conclude that although both fields have their own specific domains of interest, they share common technological developments that tend to be initiated by new developments in biotechnology and computer science.

## Background

The SYMBIOmatics Specific Support Action (SSA) is a European funded project. The main goal is to identify synergies between the bioinformatics (BI) and medical informatics (MI) research domains. In addition to experts that are approached through a survey, input will also be gathered from the analysis of scientific literature. In this paper, we focus on the analysis of scientific literature.

Bioinformatics (BI) and medical informatics (MI) are two research fields that have become mature in the past 20 years. They serve the needs of different but related research communities: BI provides solutions to scientists doing biological research whereas MI fulfils the demands from clinical personnel, for practitioners and scientists in medical research [1,2]. Although biological research may be part of a medical research project, it is often unclear how BI and MI research are coupled together [3]. Both research domains profit from progress in new IT developments and computer science as well as related scientific fields (e.g. physics, mathematics, etc.). However, the degree of exchange of new developments between the BI and the MI research domain has not been analysed [4].

Some indications of cross-fertilisation between the BI and the MI domain have been reported [5]. Both domains share a common IT infrastructure (e.g. electronic databases and terminologies), and scientists in both domains adopt solutions from the other domain if they work in an interdisciplinary environment (e.g. biological research done in a clinical environment) [6]. Last but not least, both domains share the common goal to provide new IT-based solutions to biomedical research and contribute to the treatment and cure of diseases. As a result synergies between MI and BI research can be expected as they contribute to medical or biological research that aims at a better understanding of the molecular basis of diseases, i.e. the genetic predisposition for a disease [7].

Although BI and MI contribute to biomedical research and share information technology, the extent to which researchers in the BI domain contribute to ongoing work in MI research and vice versa has not yet been analysed. Some researchers will be active in both fields, i.e. they collaborate with researchers from the BI and the MI domain and publish in journals reporting on MI research as well as in journals for BI research. A different indicator of cross-fertilization between both domains is the uptake of new technologies from the other domain, e.g. postprocessing of data from microarray experiments and the use of controlled vocabularies such as UMLS and gene ontology. Although it can be expected that BI and MI researchers benefit from common research, development and collaborations, it is yet unclear to which extent researchers are active in both fields and how current and future collaborations can lead to benefits for both sides. Therefore we analyzed a large set of publications from BI and MI research to identify topics that are relevant to both research domains.

The scientific literature forms the repository of research accomplished in the past. Medline provided by the National Library of Medicine (NLM, Bethesda, MD, U.S.A) is the most comprehensive set of documents of biomedical research covering BI and MI research as well. Each Medline abstract contains in a condensed form details on technologies applied and results obtained. As part of the SYMBIOmatics project abstracts from BI and MI journals were processed to extract topics that are shared between the BI and MI domain and thus have the potential for synergies for both.

In recent years Medline abstracts have been used to extract facts such as protein-protein interactions, functional annotations of proteins, pathway information, point mutations, gene-disease associations and other protein or gene related information [8-11]. All approaches rely on existing terminological resources to extract facts from the literature that are linked to the known terms. It is obvious that there is no terminological resource representing all BI and MI topics. By contrast every new scientific publication could contain a new topic depending on the potential of the solution presented in the document. Others have proposed to extract paradigm shift patterns from the text, but rely on known syntactical patterns for the representation of such facts [12]. Such patterns are not available for new emerging technologies or for common topics between the BI and the MI domain. The identification of microparadigms, i.e. chains of collective reasoning, and discourse structure in the documents is as well not suitable, since new emerging technologies are not part of a discourse structure [13,11]. As a result we chose to analyze the distribution of bigrams from the literature to find evidence for new emerging technologies in the literature.

The rest of the document is organized as follows. The "Result" section reports on identified and shared topics between both domains and in the "Discussion" section we interpret the findings and discuss shortcomings of our approach. In the "Method" section we describe the generation of the corpus and the extraction of bigrams.

## Results

The BI journal corpus contains 8,696 documents and the MI journal corpus 6,309 documents (table 1). The BI query corpus consists of 142,656 documents in comparison to 49,119 documents in the MI query corpus; 689 documents were in both corpora (not shown). Comparing statistical parameters describing the BI journal corpus and the MI journal, we find that the size of both corpora and the distribution of bigrams extracted from both corpora are similar (table 1).

Analyzing the overlap between the BI query corpus and the two journal corpora shows that 44% of the BI journal corpus is contained in the BI query corpus whereas only 3% of the MI journal corpus overlaps with the BI journal corpus (table 2). 62% of the MI journal corpus overlaps with the MI query corpus, but only 8% of the BI journal corpus. The MI journal corpus seems to be more homogeneous than the BI journal corpus and better represented in the MI query corpus in comparison to the two corpora for the BI domain.

**Figure 1:**
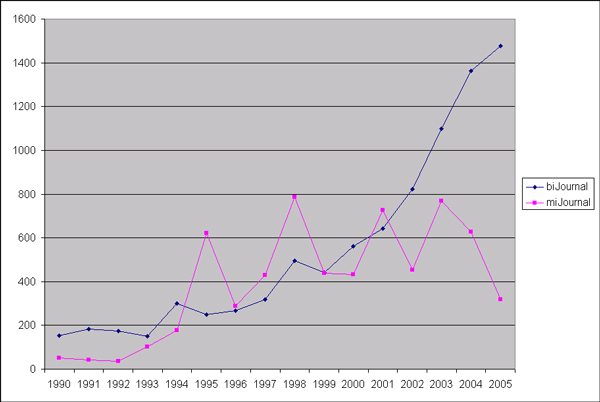
**Sample figure title**. Distribution of the publications from the BI journal corpus and from the MI journal corpus over time. The number of publications continuously increased BI domain a continuous increase in publications in the used journals took place. In the MI field the number of publications fluctuated over time, which might be the result of conferences that did not take place every year. Relative low publication figures in 2005 are partly due to the fact that not all publications of 2005 have yet been incorporated into the Medline distribution. Nevertheless, the publication number in 2005 in the BI field showed already an increase in published articles, which could be the result of open access publishing.

We extracted the publication date of the documents from the BI journal corpus and the MI journal corpus and calculated the distribution over time (figure 1). We observe a strong increase in publications in the BI field over the past 5 years, whereas the main growth in publications in the MI field took place during 1990 and 2000. In the case of the BI journal corpus the most frequent bigrams over the past 15 years are "gene expression" (Df = 711), "amino acid" (Df = 490) and "protein sequence" (Df = 438; table 3). In the same way the selection of the most frequent bigrams from the MI journal corpus ("information system", Df = 899; "health care", Df = 881; and "decision support", Df = 536; table 4) has again the same distribution as known across the whole document set. We conclude that researchers working on the most relevant topics to the MI and the BI domain generate a continuous stream of publications for every journal and conference of the domain.

For the identification of new technologies and topics we identified those bigrams that have been mentioned during the period 2000–2005 but at a low frequency before (called "emerging bigrams"). From all emerging bigrams we selected the15 bigrams with the highest document frequency and compared them to bigrams that had the highest document frequency amongst recent and past documents. In the BI journal corpus, most frequent emerging bigrams were "microarray datum" (emerged 2000, Df = 268), "microarray experiment" (2000, Df = 184) and "microarray data" (2000, Df = 169). The first bigram is already amongst the highest ranking bigrams during 2000–2005 (position 12) and is more frequent than bigrams having the ranks 8–10 for the bigrams from the past 15 years. The importance of microarray experiments for the BI domain is reflected in the high frequency of publications attached to this emerging technology and in addition by other bigrams in the list of the top 15 (e.g. "expression profile", "cdna microarray", "microarray technology" and "microarray gene"). Other topics that had a strong representation in recent documents are "gene ontology", "support vector" and "vector machine", "protein interaction" and "interaction network", "whole genome" and "nucleotide polymorphism".

Top ranking emerging bigrams in the MI journal corpus were "patient safety" (Df = 64), "gene expression" (Df = 44) and "medical error" (Df = 41). The frequency of the emerging bigrams was much lower than the frequency of the top ranking emerging bigrams in the BI journal corpus and much lower than the frequency of bigrams in recent and past documents. This shows that new developments emerged in the MI domain at a lower frequency in recent documents than in the BI domain. A few bigrams such as "gene expression", "open source" and "expression datum" are typically attributed to the BI domain. Other bigrams such as "support vector" and "vector machine" show that the MI domain as well as the BI domain profit from new developments in computer science and mathematics.

We extracted all bigrams with high TfIdf values that emerged between 2000 and 2005, i.e. all bigrams that were not mentioned before 2000 and that had a high frequency in the corpus. As expected microarray experiments and technologies related to microarrays were the most prominent developments starting in 2000 (table 5). Other emerging new topics refer to "gene ontology", "support vector" and "vector machines", "text mining", "open source", "system biology", "association study" and other. From 2002 to 2003 new topics are again related to microarray experiments such as "false discovery" and "discovery rate", "r package" and "microarray study", whereas others are related to ontologies ("go term", "go annotation"). During this period and during 2004–2005 new topics refer to splicing ("splicing event") and text mining ("biocreative task", "task 1a", "task 2").

In the MI domain new topics between 2000 and 2001 emerged at a lower frequency (TfIdf value). In synergy to the BI domain, the topics "open source", "expression datum", "support vector" and "vector machine" emerged (table 6). In contrast to the BI domain the topics "medical error", "snomed ct" and "study background" were prominent. During 2002 to 2003 bigrams related to microarray technology appeared as well as the topic "gene ontology", all are primarily attributed to the BI domain, but not necessary originated in the BI domain. In the past 2 years in particular "grid technology" and "ubiquitous computing" as well as tissue microarray data exchange specification ("tma des", "microarray data", "exchange specification") emerged.

Altogether, a number of topics are shared between the BI and the MI domain that have developed over the past 5 years (microarray experiments, ontologies, open source, text mining, support vector machines). All of them are the basis of synergetic development.

## Discussion

Both the BI and the MI domain undergo fast changes: new biomedical and IT technologies are introduced and lead to changes in research. The rate of publications in the BI domain shows a strong increase over past years with a large portion of the research work directly linked to microarray experiments. The importance of microarray experiments for biomedical research is also visible in the MI domain and will become a lot more visible in the MI domain in the future.

In the MI domain, "patient safety" and "medical error" were strong emerging topics reflecting concerns resulting from recent studies that errors in medical treatment could be avoided with better IT support [14]. By nature these topics will not be of any importance to the BI domain.

Synergies between the BI and the MI domain exist for several reasons. First, both domains profit from new biomedical developments such as microarray technology. Second, both domains profit from new developments in computer science and mathematics (e.g. support vector machines). Third, new topics and developments in the BI domain had in the past an influence on the MI domain such as gene ontology and open source development or software. Last both BI and MI profit from ongoing developments that take place at the same time in either domains (e.g. text mining and ontologies) [15].

Gene ontology and microarray experiments became frequent in the BI domain around 2000 whereas the same topics emerged in 2002 in the MI domain. This is a short time period taking into consideration that generating and publishing of research results takes time. We conclude that in principle relevant research results between both domains are exchanged at a fast rate, but they might not be relevant right away. It is an open question whether "association study", "haplotype block" and "system biology" from the list of new bigrams in the BI domain (table 5) will reappear amongst the new bigrams in the MI domain in the near future. On the other side, it is surprising that "grid technology" does not have a high frequency in the BI domain and that "marker gene" does not appear in the list of new bigrams in the MI domain.

Finally it is obvious that not all emerging topics could be identified in our analysis since it relies on the extraction of bigrams. New topics that have not been identified are telemedicine, pharmacogenomics, biochips and lab-on-a-chip.

## Conclusion

From our analysis of the scientific literature for bioinformatics and medical informatics we find that although both fields have their own specific domains of interest, they share common topics. The analysis of microarray experiments as a shared topic is driven by the new technology changing biological and medical research. Other topics such as text mining and ontology development is co-evolving in both domains and support vector machines have been introduced to both domains at the same time by new developments in computer science and mathematics. These topics form currently the core of synergies between the BI and the MI according to our literature analysis. It could happen that new topics currently relevant to the BI domain and related to population genetics and system biology will be more prominent in the near future.

## Methods

Four corpora were extracted from EBI's inhouse installation of Medline (Release date 25^th ^November 2005). All documents were published during the period 1990 to 2005. The first and second corpus consist of Medline abstracts belonging to publications in BI journals and MI journals, respectively (called BI journal corpus and MI journal corpus, table 1). The following journals were selected.

1. BI journal corpus: Bioinformatics, Biosystems, BMC Bioinformatics, Brief Bioinform, Comput Methods Programs Biomed, IEEE Trans Inf Technol Biomed, J Bioinform Comput Biol, J Biomed Inform, J Comput Aided Mol Des, J Comput Biol, Pac Symp Biocomput

2. MI journal corpus: AMIA Annu Symp Proc, Artif Intell Med, BMC Med Inform Decis Mak, Int J Med Inform, J Am Med Inform Assoc, Medinfo, Methods Inf Med, Proc AMIA Symp

The other two corpora consisting of Medline abstracts retrieved by keyword queries served as reference sets. The queries have been applied to both the MeSH terms and the content of the Medline abstract. Queries consisted of bioinformatics keywords and of medical informatics keywords, called BI query corpus and MI query corpus, respectively (ref. to additional file 1 for applied query terms).

All corpora were separated into two sets: the first one covering the years 2000 to 2005 ("recent documents") and the second one covering the years 1990 to 1999 ("past documents"). All corpora were processed in the same way using a modular information extraction infrastructure available from the European Bioinformatics Institute [20]. The compute server was a Linux farm of 220 IBM dual-cpu nodes (1.2–2.8 Ghz, 2 GB RAM).

The noun phrases were selected from the documents, where a noun phrase is represented by the language pattern "Det (Adj|Adv|Noun)+ Noun+". All noun phrases were processed to extract all contained bigrams, which then serve as features of the document representing the content. A bigram is any combination of two consecutive words from the noun phrase. The leading determiner was dropped. Every word of the noun phrase was normalized to lower case and lemmatized to use the base form only. For example, the noun phrase "the protein secondary structure" was split up into the noun phrases "protein secondary" and "secondary structure". Every Medline abstract was represented by a list of bigrams extracted from the document.

The extraction of bigrams from noun phrases is advantageous in comparison to the use of single terms from noun phrases, since single terms tend to be ambiguous. On the other side, bigrams are less specific than noun phrases, since bigrams are shorter and have less syntactical variability.

For every bigram, the frequency in the document was calculated (term frequency, Tf) as well as the frequency of the bigram in all documents of the corpus (document frequency, Df) resulting in the TfIdf value (Tf / Df) for every bigram. Recent and past documents were processed separately. For every document, the bigrams were ranked according to their TfIdf value and the 10 bigrams with the highest TfIdf score were selected for further analysis. Note that some documents do contain bigrams that have only a relatively low TfIdf score in comparison to the whole set of all identified bigrams. Such documents either deal with new developments or with a niche research topic. These bigrams were also included into the analysis, since they represent a document. If bigrams were mentioned in less than 20 documents over the period from 1990 to 2005, then they were excluded from further analysis. All bigrams were again ranked according to their TfIdf value.

We computed 2 bigram lists for each of the 4 corpora: one list contained the bigrams for the recent documents and the other for the past documents. We extracted from the bigram list of the recent documents all the bigrams that were mentioned amongst the bigrams of the past documents at a very low document frequency (Df < 4) and which had a high Df score after 1999, which resulted in the list of "emerging bigrams". Any bigram not mentioned before a given time period is called a "new bigram".

## Authors' contributions

All authors have worked together on the SYMBIOmatics project and have contributed to the discussion on the synergies between the BI and MI research domains. The literature analysis is the result of collaborative work between Dietrich Rebholz-Schuhmann, Erik van Mulligen, Jean-Louis Coatrieux and Johan van der Lei and was delivered as a result of a working package to the project. Special thanks belong to Graham Cameron, Dominic Clark, Fernando Martin-Sanchez and Luciano Milanesi for feedback on the manuscript and for integration of the analysis into the consultation work of the project.

## Supplementary Material

Additional file 1 Click here for file

## Figures and Tables

**Table 1:** Number of bigrams in the BI and MI corpora.

	2000 – 2005				1990 – 1999		
	
	documents	bigrams	bigrams	bigrams	documents	bigrams	bigrams
		all	Df > 20	Df > 20		all	Df > 20
				emerging			
BI journal corpus	5,968	12,992	701	172	2,728	10,777	119
BI query corpus	90,082	50,248	15,406	4,666	52,574	33,438	8,862
MI journal corpus	3,330	8,604	257	15	2,979	8,569	186
MI query corpus	21,609	34,432	2,284	60	27,510	44,043	2,463

4 different sets of Medline abstracts were analyzed (ref. to text). All documents were categorized as recent documents (2000 – 2005) and past documents (1990 – 1999). From all documents bigrams were extracted from noun phrases (for details see text). The analysis was restricted to bigrams with document frequency of at least 20. In the set of recent documents we identified those bigrams that were not mentioned before 2000 ("emerging"). The BI journal corpus and the MI journal corpus are similar in terms of the document members and contained bigrams.

**Table 2:** Overlap between the query and the journal corpora.

	BiQueryCorpus (142,656 docs)	MiQueryCorpus (49,119 docs)
BiJournalCorpus (8,696 docs)	3,837	731
MiJournalCorpus (6,309 docs)	215	3,925

The table displays the number of Medline abstracts contained in four corpora extracted from Medline (ref. to text). As expected there is a strong overlap between the BI journal corpus and BI query corpus and between the MI journal corpus and MI query corpus. The intersection between BI journal corpus and MI query corpus is small as well as the intersection between MI journal corpus and BI query corpus. This shows that the selection of the corpora based on the journal titles already leads to a selection of documents that represent information for the BI domain which is different from the MI domain. In the case of the BI journal corpus less than half of the documents are contained in the BI query corpus. This finding indicates that the query terms for the BI query corpus might be still too restrictive to cover the whole BI domain knowledge.

**Table 3:** Emerging bigrams in the BI journal corpus.

		Rank	Rank	Rank			Rank	Rank	Rank
	Df	emerging	2000–2005	1990–1999		Doc. Freq.	emerging	2000–2005	1990–1999
gene expression	711		1	1	microarray experiment	184	2	22	
amino acid	490		2		not only	181		23	15
protein sequence	438		3	2	microarray data	169	3	25	
expression datum	339		4		expression profile	168	4	26	
sequence alignment	321		5		gene ontology	135	5	37	
supplementary information	321		6		support vector	133	6	38	
sequence alignment	321			3	vector machine	130	7	41	
dna sequence	313		7	4	protein interaction	99	8	62	
protein structure	313		8	5	whole genome	80	9	74	
freely available	306		9		nucleotide polymorphism	76	10	80	
binding site	295		10	6	cdna microarray	73	11	83	
large number	288		11	7	microarray technology	73	12	84	
microarray datum	268	1	12		microarray gene	66	13	85	
neural network	250		13	8	data mining	60	14	87	
secondary structure	246		14	9	interaction network	60	15	88	
new method	244		15	10					
data set	236		16	11					
datum set	224		17	12					
source code	208		18	13					
markov model	187		21	14					

The table shows bigrams extracted from the BI journal corpus (col. 1) together with their document frequency (col. 2) and their ranks. The first rank refers to emerging bigrams (ref. to text, col. 3), the second rank is for bigrams with their highest document frequency during 2000–2005 (col. 4) and the last rank uses the highest document frequency during 1990–1999 (col. 5). The table shows that over the last five years new topics at a high frequency emerged.

**Table 4:** Emerging bigrams in the MI journal corpus.

		**Top 15 rank**					**Top 15 rank**		
	**Doc. Freq.**	**emerg-ing**	**2000–2005**	**1990–1999**		**Doc. Freq.**	**emerg-ing**	**2000–2005**	**1990–1999**
information system	899		1	1	patient safety	64	1	75	
health care	881		2	2	gene expression	44	2	87	
decision support	536		3	3	medical error	41	3	92	
medical record	445		4	4	digital assistant	35	4	94	
patient record	427		5	5	personal digital	35	5	95	
medical informatics	397		6	6	disease management	31	6		
clinical information	330		7	7	open source	28	7		
health information	294		8		provider order	25	8		
patient care	285		9	8	clinical documentation	23	9		
support system	284		10	9	clinical document	23	10		
electronic medical	261		11		support vector	23	11		
information technology	245		12		vector machine	22	12		
clinical practice	210		13	10	expression datum	21	13		
medical information	203		14		study objective	21	14		
neural network	203			11	snomed ct	20	15	100	
knowledge base	198		15						
natural language	196			12					
clinical datum	194			13					
hospital information	191		16	14					
electronic patient	180			15					

The table shows bigrams from the MI journal corpus (ref. to table 1 for details). The table shows that emerging topics played only a minor role in recent documents.

**Table 5:** New bigrams in the BI journal corpus in recent years.

**New in 2004–2005**	**Df**	**New in 2002–2003**	**Df**	**New in 2000–2001**	**Df**
protein background	16	false discovery	41	microarray datum	268
method conclusion	12	discovery rate	40	microarray experiment	183
annotation method	11	datum background	40	expression profile	168
dataset result	11	microarray study	36	microarray data	161
array cgh	10	text mining	35	gene ontology	135
protein localization	10	association study	28	support vector	133
organism database	10	r package	26	vector machine	130
ontology database	10	normalization method	25	protein interaction	99
biocreative task	9	multiple testing	23	nucleotide polymorphism	76
entity recognition	9	ontology term	22	cdna microarray	73
splicing event	8	go term	21	microarray technology	73
name recognition	8	gene list	20	microarray gene	65
lowess normalization	8	human protein	20	differential expression	59
anatomy ontology	7	biomedical text	19	open source	54
novo sequencing	7	complex disease	19	biological network	50
task 2	6	microarray result	18	microarray analysis	48
task 1a	6	homo sapiens	18	widely used	48
venn diagram	4	named entity	17	gene selection	46
database identifier	4	synonymous codon	16	interaction datum	37
		gene clustering	16	system biology	34
		mammalian genome	16	interacting protein	33
		bioinformatics analysis	15	alternative splicing	32
		haplotype block	14	oligonucleotide microarray	29
		go annotation	13	related gene	27
		two dataset	13	web application	27
		expression result	13	biological sample	26
		marker gene	12	expression value	23
		dimensionality reduction	12	primer design	22

The table shows bigrams from the BI journal corpus that were new during the period 2004–2005 (col. 1 and 2), the period 2003–2004 (col. 3 and 4) and the period 2000–2001 (col. 5 and 6). All bigrams were selected and ranked according to their document frequency value (ref. to text), which had to be above 3. During the time 2000–2001 a large number of bigrams referring to microarray experiments emerged. "task 1a" and "task 2" are exclusively linked to BioCreAtive. "false discovery" refers to false discovery rate (FDR) in DNA microarray analysis.

**Table 6:** New bigrams in the MI journal in recent years.

**New in 2004–2005**	**Df**	**New in 2002–2003**	**Df**	**New in 2000–2001**	**Df**
grid technology	10	microarray experiment	14	medical error	41
computation time	7	microarray datum	14	open source	28
grid service	6	dna microarray	13	support vector	23
grid objective	6	Microarray data	12	vector machine	22
grid infrastructure	5	computerized provider	10	expression datum	21
respiratory syndrome	5	syndromic surveillance	10	snomed ct	20
gene selection	5	year 2013	10	system conclusion	19
microarray gene	4	Semantic web	10	study background	14
secondary structure	4	setting method	10	patient method	12
hierarchical clustering	4	expression level	9	medication order	12
		result conclusion	9	cpoe system	11
		cpoe implementation	8	search tool	11
		gene ontology	8	method conclusion	11
		System functionality	8	patient empowerment	10
		mobile phone	7	search filter	10
		exclamation mark	7	partner healthcare	10
		inverted exclamation	7	detection system	10
		ubiquitous computing	6	intermountain health	10
		online evidence	6	guideline element	10
		health literacy	6	overall goal	10
		expression profile	6	xml schema	9
		Electronic prescribing	6	original study	9
		wireless handheld	5	snomed clinical	9
		pda use	5	exploratory study	9
		digital pen	5	informatics method	9
		computational modeling	5	hl7 rim	9
		collaborative clinical	5	mesh thesaurus	9
		evidence system	4	search method	9
				online health	8
				Functional magnetic	8

The table shows bigrams that were extracted from the MI journal corpus. Again all bigrams are mentioned first in the years 2000 to 2005 (ref. to Table 5). New technologies are: grid technology, tissue engineering, clinical bioinformatics, tissue microarray (tma) and TMA data exchange specification (TMA DES by the Association of Pathology Information, PMIDs 15871741 and 16086837), gene ontology and semantic Web. "Year 2013" refers to a set of publications related to the subject "Quo vadis Health care" (PMIDs 1245355{2,4,5,6,7,8,9}, 1245356{1,4,5}).
