# Supplementary material for: SYMBIOmatics: Synergies in Medical Informatics and Bioinformatics – exploring current scientific literature for emerging topics
Source: BMC Bioinformatics. 2007 Mar 8;8(Suppl 1):S18. doi: 10.1186/1471-2105-8-S1-S18 (PMC1885847; doi:10.1186/1471-2105-8-S1-S18)
Supplement: Additional file 1 [file 1471-2105-8-S1-S18-S1.doc]

List-1: Query terms to retrieve Medline abstracts linked to MI. Query

was performed on MeSH term annotations of abstracts as well as to the

Medline abstract content (Erasmus University Rotterdam)

Ambulatory Care Information System, BedSide Computing, Clinical,

Clinical Informatics, Clinical Laboratory Information Systems, Clinical

Pharmacy Information Systems, Community Care Networks, Community Health

Networks, Community Networks, Computer-Assisted, Computer-Assisted,

Computer-Assisted, Computer-Assisted, Computer-Assisted,

Computer-Assisted, Computerized, Computerized, Computerized Patient

Records, Decision Aids, Decision Analysis, Decision Making, Decision

Modeling, Decision Support, Decision Support Systems, Decision Support

Systems, Decision Support Techniques, Decision Theory, Decision Trees,

Diagnosis, Drug Therapy, Emergency Care Information Systems, Expert

Systems, Hospital Information Systems, Integrated Academic Information

Management Systems, Integrated Advanced Information Management Systems,

Knowledge Acquisition (Computer), Knowledge Bases (Computer), Knowledge

Representation (Computer), Management, Medical Decision Making, Medical

Records, Medical Records Systems, Metathesaurus, Models, Protocol Drug

Therapy, Reminder Systems, Therapy, Unified Medical Language System

List-2: Query terms to retrieve Medline abstracts linked to BI. Query

was performed on MeSH term annotations of abstracts as well as to the

Medline abstract content* (Erasmus University Rotterdam)

Bioinformatics, Computational Biology, Database, DNA Data Bank of Japan,

DNA Sequence Databases, European Molecular Biology Laboratory Nucleotide

Sequence, GenBank, Genetic, Genetic Databases, Genetic Sequence

Databases, Nucleic Acid, Nucleic Acid Databases, Nucleic Acid Sequence

Databases, Online Mendelian Inheritance in Man, Protein, Protein

Databases, Protein Sequence Databases, Protein Structure Databases, RNA

Databases, RNA Sequence Databases, SWISS-PROT
